# Lateralization Disruption and Dynamic Balance Alterations in Alzheimer's Disease: Impacts on Hemispheric Interaction and Cognitive Performance

**DOI:** 10.1002/hbm.70411

**Published:** 2025-11-14

**Authors:** Juan Wang, Yuxin Li, Yu Yang, Zhenhu Liang, Ping Xie, Xiaoli Li, Yina Guo, Shaobao Li, Xiaoling Chen

**Affiliations:** ^1^ School of Electrical Engineering Yanshan University Qinhuangdao China; ^2^ Key Laboratory of Intelligent Rehabilitation and Neuromodulation of Hebei Province Qinhuangdao China; ^3^ State Key Laboratory of Cognitive Neuroscience and Learning Beijing Normal University Beijing China; ^4^ School of Electronic Information Engineering Taiyuan University of Science and Technology Taiyuan Shanxi China; ^5^ Shanxi Provincial Sports Health Intelligent Equipment Technology Innovation Center Taiyuan Shanxi China

**Keywords:** Alzheimer's disease (AD), dynamic lateralization, dynamical balance, laterality fluctuation (LF), laterality reversal (LR)

## Abstract

Brain lateralization is considered evolutionarily adaptive. Impaired functional specialization is thought to cause abnormal lateralization in neurological disorders. However, the dynamic changes in brain laterality in Alzheimer's disease (AD) remain unclear. In this study, resting‐state fMRI data and neuropsychological assessments from 109 participants (49 ad patients and 60 healthy controls [HC]) were used. Dynamic laterality time series were constructed by extracting the dynamic laterality index (DLI) within each sliding window. We assessed two key features: laterality reversal (LR), reflecting intra‐hemispheric processing efficiency, and laterality fluctuation (LF), indicating inter‐hemispheric communication. Group differences in dynamic laterality characteristics were analyzed using statistically rigorous methods, regressing out gender, age, years of education, and head movements. Spearman correlation analyses examined the relationship between laterality characteristics and cognitive performance. Our results showed that AD patients exhibited a more pronounced loss of left lateralization as well as stronger right lateralization, especially in the somatomotor network (SMN) and default mode network (DMN). Additionally, we observed decreased LR as well as increased LF with global trends in AD. These divergent changes disrupted the dynamical balance between intra‐ and inter‐hemispheric information interaction. Notably, this imbalance depended on the degree of lateralization, and the higher order cognitive networks with high‐level lateralization were more vulnerable. Importantly, the observed abnormal lateralization metrics were associated with worse cognitive impairment. Our study highlights a disruption of dynamic lateralization balance in higher order cortical networks in AD patients and reveals its potential role in the disease's pathophysiology.

## Graphical abstract

We employed dynamic laterality index (DLI) analysis of resting‐state fMRI to quantify laterality reversal (LR) and laterality fluctuation (LF) in patients with Alzheimer's disease (AD). The results revealed a disrupted dynamic balance between LR and LF, along with altered hemispheric dominance, which was associated with cognitive impairment in AD.

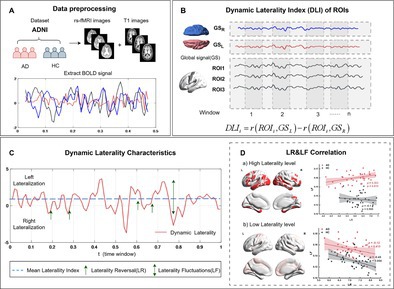

**Summary**

- AD patients show diminished left‐hemisphere and heightened right‐hemisphere lateralization—especially in SMN and DMN—likely driven by gray‐matter asymmetry loss and corpus callosum degeneration.
- AD disrupts the dynamic balance of lateralization through a global reduction in intra‐hemispheric processing and an increase in inter‐hemispheric communication, making highly lateralized, higher‐order networks such as the DMN and DAN particularly vulnerable to this imbalance.
- Abnormal dynamic lateralization metrics strongly correlate with cognitive decline, making them promising biomarkers and early intervention targets in AD.

**Abbreviations**
AD: Alzheimer's disease
ADAS‐Cog: Alzheimer's Disease Assessment Scale‐ Cognitive Subscale
CON: control network
CSF: cerebrospinal fluid
DAN: dorsal attention network
DefaultA_PCC: posterior cingulate cortex
DefaultA_PFCd: dorsal prefrontal cortex
DefaultC_Rsp: retro splenial cortex
DLI: dynamic laterality index
DMN: default mode network
fMRI: functional magnetic resonance imaging
GS: global signal
HC: healthy controls
LF: laterality fluctuation
LIM: Limbic
LR: laterality reversal
MLI: means laterality index
MMSE: Mini–Mental State Examination
ROI: Region of interest
SMN: somatomotor network
SomMotA: somatomotor network
SomMotB_Cent: precentral gyrus
SomMotB_S2: secondary somatosensory cortex
SVAN: salience/ventral attention network
TPN: temporal parietal network
VIS: visual network
WM: white matter

## 1 Introduction

Alzheimer's disease (AD) is a complex and progressive neurodegenerative disorder that has become one of the most pressing public health challenges worldwide. It is characterized by a gradual decline in cognitive abilities, particularly affecting memory, language, and executive functions. The increasing prevalence of AD, particularly among aging populations, creates the urgent need to understand its underlying mechanisms and identify effective therapeutic strategies (Tiwari et al. 2019). Cognitive decline in AD can be attributed to a range of factors, including neurodegeneration, synaptic dysfunction, and altered brain network dynamics (Zhang et al. 2024; Serrano‐Pozo et al. 2011). Together, these factors disrupt the brain's capacity to perform specialized cognitive tasks, ultimately resulting in a significant reduction in overall cognitive function. Hemispheric lateralization plays a critical role in these specialized cognitive processes by allowing the left and right hemispheres to manage distinct functions, thereby optimizing cognitive performance (Hartwigsen et al. 2021). Consequently, exploring the role of hemispheric lateralization in AD may provide valuable insights into the mechanisms driving cognitive dysfunction and contribute to the development of more effective interventions.

Hemispheric lateralization is a fundamental aspect of cognitive processing that enables the brain to specialize in certain functions, thereby enhancing cognitive efficiency. In healthy individuals, this specialization is evident: the left hemisphere predominantly governs language and verbal tasks, while the right hemisphere is more involved in spatial awareness and attentional processes (Gotts et al. 2013; Hugdahl 2000; McGraw et al. 2001; Hund‐Georgiadis et al. 2001). This division of labor not only optimizes cognitive processing but also facilitates the brain's ability to manage complex tasks in a streamlined manner. However, emerging research suggests that this delicate balance is often disrupted in individuals with AD, leading to significant cognitive impairments (Ng et al. 2021; Babiloni et al. 2018). Studies have shown that AD patients frequently exhibit a marked reduction in leftward lateralization across various cognitive networks, particularly in the default mode network (DMN) and the dorsal attention network (DAN). This decline in lateralization may reflect the neurodegenerative processes that underlie AD, progressively impairing the brain's ability to efficiently allocate cognitive resources (Liu et al. 2018; Banks et al. 2018). As a result, individuals with AD may struggle with language processing and attentional control due to diminished specialization between the hemispheres, which ultimately affects their capacity to perform everyday tasks. Despite these findings, most existing research has focused on static measures of lateralization, which fail to capture the dynamic nature of brain activity and its fluctuations during cognitive tasks.

The dynamic brain network perspective underscores that functional connectivity fluctuates over time, with networks continually adapting to shifting cognitive demands and environmental factors (Wang et al. 2014; Preti et al. 2017). This dynamic flexibility is essential for optimal cognitive performance, allowing the brain to reconfigure itself in response to different tasks and challenges (Gonzalez‐Castillo and Bandettini 2018). Crucially, such time‐varying properties cannot be captured by traditional static lateralization measures (Seghier 2008; Deppe et al. 2000), underscoring the unique value of dynamic metrics in characterizing brain organization. However, in AD, these dynamic processes are often disrupted, particularly in key left‐hemispheric regions such as the precuneus, frontal pole, and orbitofrontal cortices (Zhao et al. 2022), which are implicated in cognitive functions such as memory, attention, and executive control. This disruption likely impairs the brain's ability to process and integrate information across regions. Consequently, both cognitive and functional deficits emerge. Specifically, alterations in lateralization are thought to impair both intra‐hemispheric processing (the ability of a hemisphere to handle specialized tasks efficiently) and inter‐hemispheric communication (the exchange of information between hemispheres), both of which are crucial for effective cognition (Wu et al. 2022). The imbalance between hemispheres could undermine the ability of the brain to maintain coherent and efficient communication, thereby affecting tasks that require the integration of information from multiple brain regions. Despite these advances, it remains poorly understood how such imbalances manifest specifically as abnormal dynamic lateralization in patients with AD.

In this study, we aimed to investigate the dynamic characteristics of hemispheric lateralization in AD patients, with a focus on the interplay between intra‐hemispheric processing and inter‐hemispheric communication. Using resting‐state functional magnetic resonance imaging (fMRI), we compared dynamic patterns in networks including the DMN, SMN, and DAN, between AD patients and healthy controls (HCs). We also examined the lateralization reversal (LR) correlation with laterality fluctuation (LF) across different levels of lateralization. We hypothesized that AD would feature rightward laterality in the brain networks, especially in DMN and SMN. Both dynamic characteristics, that is, LR and LF, as well as the correlation between them would be damaged in the AD patients, and the correlation would be more vulnerable in the regions of interest with low laterality level. We also predicted that abnormal changes in lateralization patterns and correlations between the LR and LF would be associated with cognitive impairment.

## 2 Materials and Methods

### 2.1 Participants

The data used in this study were obtained from the Alzheimer's Disease Neuroimaging Initiative (ADNI; https://adni.loni.usc.edu/) (Weiner et al. 2013). Launched in 2003, the ADNI is a public‐private partnership led by Principal Investigator Michael W. Weiner, M.D. The primary goal of the ADNI is to test whether clinical and neuropsychological assessments, serial magnetic resonance imaging (MRI), positron emission tomography (PET), and other biomarkers can be combined to measure the progression of mild cognitive impairment (MCI) and early AD.

One hundred and twenty‐seven participants in this study were divided into the HC (*n* = 71) and AD (*n* = 56) groups. Informed consent was obtained from all participants or their authorized representatives, and the study procedures were approved by the Institutional Review Boards of all participating ADNI centers. The HC participants were required to have Mini–Mental State Examination (MMSE) scores of 26 or above, and AD patients had MMSE scores between 16 and 26 (inclusive). Subsequently, participants were excluded if their functional scans exhibited excessive motion (see Section 2.2 for criteria), leaving 60 HC participants and 49 patients with AD for further analysis. These two groups were well matched on key demographic factors, including age, gender, ethnicity, and handedness. Detailed demographic and neuropsychological profiles are summarized in Table 1.

**TABLE 1:** Demographics and clinical information.

Characteristics	HC (*n* = 60)	AD (*n* = 49)	*p*
Age	73.86 ± 7.64	74.94 ± 7.51	0.521
Gender (M/F)	24/36	27/22	0.345
Education (years)	16.58 ± 2.27	15.49 ± 2.44	0.161
Handedness (R/L/A)	58/2/0	48/1/0	0.759
MMSE	29.13 ± 1.14	21.76 ± 2.70	0.000***
ADAS‐Cog	8.15 ± 3.46	34.44 ± 8.97	0.000***

*Note:* Continuous variables are displayed as the mean ± standard deviation. *** indicates a significance of *p* < 0.001.

Abbreviations: AD, Alzheimer's disease; ADAS‐Cog, Alzheimer's Disease Assessment Scale‐Cognitive Subscale; HC, healthy control; M/F, male/female; MMSE, Mini‐Mental State Examination; R/L/A, right/left/ambidextrous.

### 2.2 Image Acquisition and Preprocessing

All participants were scanned using a 3.0‐T Philips MRI scanner. Resting‐state functional images were obtained using an echo planar imaging sequence with the following parameters: 140 volumes, repetition time (TR) = 3000 ms, echo time (TE) = 30 ms, flip angle = 80°, number of slices = 48, slice thickness = 3.3 mm, isotropic voxel size = 3.3 mm × 3.3 mm × 3.3 mm, and matrix = 64 × 64.

Both functional and structural images were preprocessed using the Data Processing Assistant for Resting‐State fMRI (DPARSF; https://rfmri.org/DPARSF/) (Yan and Zang 2010), which is based on Statistical Parametric Mapping software (SPM12; https://www.fil.ion.ucl.ac.uk/spm) (Friston et al. 2007) and Resting‐State fMRI Data Analysis Toolkit (REST; https://rfmri.org/REST) (Song et al. 2011). The fMRI data were preprocessed as follows: (1) The first 10 image volumes of the resting‐state data were discarded to ensure signal equilibrium and participants' adaptation to the fMRI scanning noise. (2) The remaining 130 images were corrected for timing differences between each slice. (3) Rigid body corrections were implemented for head motion (six‐parameter rigid body). Participants were excluded if their maximum displacement exceeded 3 mm in any direction (*x*, *y*, *z*) or if angular motion exceeded 1°. Based on these criteria, 18 participants (11 HC and 7 ad) were excluded from further analysis. (4) The realigned images were spatially normalized to the standard echo‐planar imaging template, based on the Montreal Neurological Institute stereotactic space, and then resampled into 3 mm × 3 mm × 3 mm cubic voxels. (5) Data were spatially smoothed with a Gaussian kernel of 6 mm × 6 mm × 6 mm full width at half maximum (FWHM) to decrease spatial noise. (6) Signals from white matter (WM) and Cerebrospinal Fluid (CSF), as well as Friston‐24 head motion parameters were regressed out. (7) Subsequently, linear detrending and bandpass filtering (0.01 Hz < *f* < 0.08 Hz) were applied. The global signal (GS) was not regressed out because the mean hemispheric time series were used to calculate the lateralization index (McAvoy et al. 2016). After exclusion, the mean framewise displacement (FD) across all retained participants was 0.30 mm, with a maximum FD of 0.50 mm.

### 2.3 Lateralization Metrics Computation

To assess the dynamic changes in brain lateralization of hemispheres, we utilized a sliding time window based on a GS to capture the time‐varying lateralization architecture for each region of interest (ROI) (Wu et al. 2022). The whole brain was parcellated into 114 ROIs (57 ROIs per hemisphere) using the Yeo 17‐network cortical atlas (Yeo et al. 2011). For our network‐level analysis, the original 17 networks were grouped into 8 larger functional networks. These included the DMN, control network (CON), salience/ventral attention network (SVAN), limbic (LIM), DAN, somatomotor network (SMN), visual network (VIS), and temporal parietal network (TPN) (Yeo et al. 2011). To further assess the robustness of the findings, we conducted subsequent analyses with the Schaefer atlas (Schaefer et al. 2018) (see Supporting Information). The mean fMRI time course was extracted from each ROI for each participant. We then used a window length of 20 TRs and a step of 1 TR to calculate the dynamic laterality index (DLI) for each region. For each sliding window, the DLI is defined as follows:

$${\mathrm{DLI}}_{i}=r\left({\mathrm{ROI}}_{i},{\mathrm{GS}}_{L}\right)-r\left({\mathrm{ROI}}_{i},{\mathrm{GS}}_{R}\right)$$

where ${\mathrm{ROI}}_{i}$ denotes the BOLD time series of ${\mathrm{ROI}}_{i}$, while ${\mathrm{GS}}_{L}$ and ${\mathrm{GS}}_{R}$ denote the mean global signal, which is the BOLD time series averaged across all voxels within the left and right hemispheres respectively. The parameter r refers to the Fisher‐transformed value of the Pearson correlation coefficient. We further computed the mean laterality index (MLI) for each ROI by averaging its DLI across all time windows. The MLI reflects whether the activity of a ROI is more synchronized with the left or right hemisphere. Specifically, the positive and negative MLI values indicate that an ROI is intrinsically left‐ and right‐lateralized respectively. Additionally, the intrinsic laterality for each network was defined as the sum of the MLI of all ROIs within that network. To characterize the dynamics of regional laterality, we calculated LF and laterality reversal (LR) to represent the magnitude and sign of laterality, respectively. LF is defined as the standard deviation across all windows, reflecting variability in laterality, while LR is determined by the number of zero crossings, indicating switches between left and right lateralization in consecutive windows. Together, these measures capture both moderate and extreme changes in laterality over time, and they typically demonstrate opposing associations with cognitive performance. Finally, we performed sensitivity analyses using alternative window settings to verify the robustness of our findings (see Supporting Information).

### 2.4 Associations of Laterality Fluctuation With Laterality Reversal

Given that LF and LR are postulated to capture distinct and potentially opposing aspects of dynamic laterality (Wu et al. 2022), we sought to investigate the interplay between them. To do this, we quantified their association using Spearman correlation analysis at two levels. At the individual level, the correlation values for each participant were quantified by assessing the relationship between LF and LR across all ROIs within each network. At the group level, we computed the mean spatial distributions of LF and LR for each group, resulting in a single LF‐LR correlation coefficient for each group.

To further investigate the effects of laterality on the relationship between LF and LR, the degree of lateralization was selected as the independent variable. We ranked all ROIs based on their MLI strength and categorized them into three MLI levels: high, middle, and low. Each MLI level comprised 38 ROIs (i.e., 114/3). We then computed the correlations between LF and LR for the ROIs within each MLI level. To evaluate the contributions of network‐wise laterality to these associations, we calculated the contribution of each network at a given lateralization level. This was done by dividing the ratio of the number of ROIs belonging to each network within a particular MLI level by the total number of ROIs at that level (i.e., 38) and then dividing by the weight of the respective network across the entire brain (i.e., the number of ROIs within each network divided by 114). It should be noted that due to the limited number of ROIs contained within the LIM (4 ROIs) and TPN (2 ROIs), these two networks were excluded from the subsequent analysis.

### 2.5 Statistical Analysis

Between‐group differences in demographic properties and clinical assessments were tested via the *χ* ^2^ test for categorical variables (gender and handedness) and the independent *t* test for continuous variables (age, education, MMSE, and ADAS‐Cog).

We compared each of the three dynamic lateralization metrics, including the MLI, LF, and LR, between the AD and HC groups using permutation tests. For these tests, we randomly shuffled participant group labels (AD vs. HC) and recalculated the mean group difference 10,000 times. This process generated a null distribution of group differences under the assumption that no true difference exists. The observed group difference was then compared with this null distribution to obtain the *p* value. Age, sex, years of education, handedness, and head movement (mean FD) were included as covariates to control for potential confounding effects. All resulting *p* values were further corrected for multiple comparisons using the FDR procedure, with a significance threshold set at *q* < 0.05.

To assess the relationships between dynamic laterality (LR and LF) and clinical measures, we correlated the network‐wise and ROI‐wise measures (MLI, the association between LF and LR) with the MMSE and ADAS‐Cog across all participants. The Spearman rank correlation was performed between them after controlling for the effects of gender, age, years of education, handedness, gray matter volume, WM volume, and mean FD.

## 3 Results

### 3.1 Demographic Characteristics

There were no significant differences in the demographic characteristics including age, gender, education, and handedness (Table 1). As expected, participants with AD showed lower MMSE scores and higher ADAS‐Cog scores than the HC group.

### 3.2 Static Lateralization Alterations in AD: Enhanced Inter‐Hemispheric Segregation and Rightward Laterality in the SMN and DMN

**FIGURE 1:**
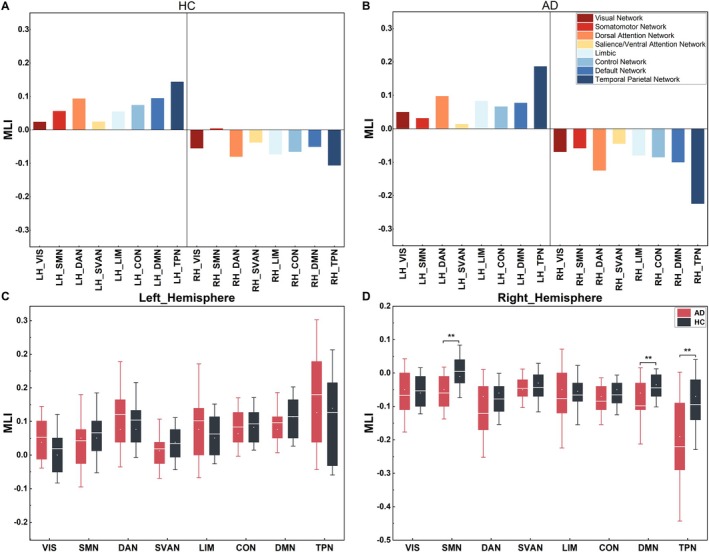
The spatial distribution of the dynamic laterality in the larger‐scale brain networks. The mean MLI values for the 16 subnetworks in the left and right hemispheres of the HC group (A) and the AD group (B). The disparities in MLI values observed between the eight networks in the left (C) and right (D) hemispheres of subjects in the AD and HC groups. ** indicates significance of *p* < 0.01. A total of 114 ROIs were divided into 16 subnetworks based on their location in the left or right hemisphere. The MLI values of the ROIs in each region were then averaged to obtain the MLI value for each of the 16 subregions. A positive MLI of a region indicates stronger interaction with the left hemisphere (leftward laterality), whereas a negative MLI indicates rightward laterality. AD, Alzheimer's disease; CON, control network; DAN, dorsal attention network; DMN, default mode network; HC, healthy control; LH, left hemisphere; LIM, limbic; MLI, mean laterality index; RH, right hemisphere; SMN, somatomotor network; SVAN, salience/ventral attention network; TPN, temporal parietal network; VIS, visual network.

The network‐wise mean lateralization indices (MLIs) were calculated for each hemisphere, resulting in eight pairs of MLIs for the bilateral hemispheres in each cohort (Figure 1). These MLIs were visually inspected and then included in further analysis. Notable discrepancies in lateralization between the left and right hemispheres were observed between AD and HC groups. Specifically, in comparison with the HC group, the AD group showed increased lateralization in both hemispheres across most networks. However, the SMN and SVAN showed reduced leftward lateralization in the left hemisphere. Notably, significant rightward lateralization was found in the AD group for the SMN (*t* = −4.25, *p* = 0.002), DMN (*t* = −2.99, *p* = 0.008), and TPN (*t* = −3.46, *p* = 0.002) in the right hemisphere (Figure 1D). These findings suggest that participants with AD had stronger intra‐hemispheric lateralization and reduced inter‐hemispheric integration, as reflected by higher MLIs in both hemispheres.

**FIGURE 2:**
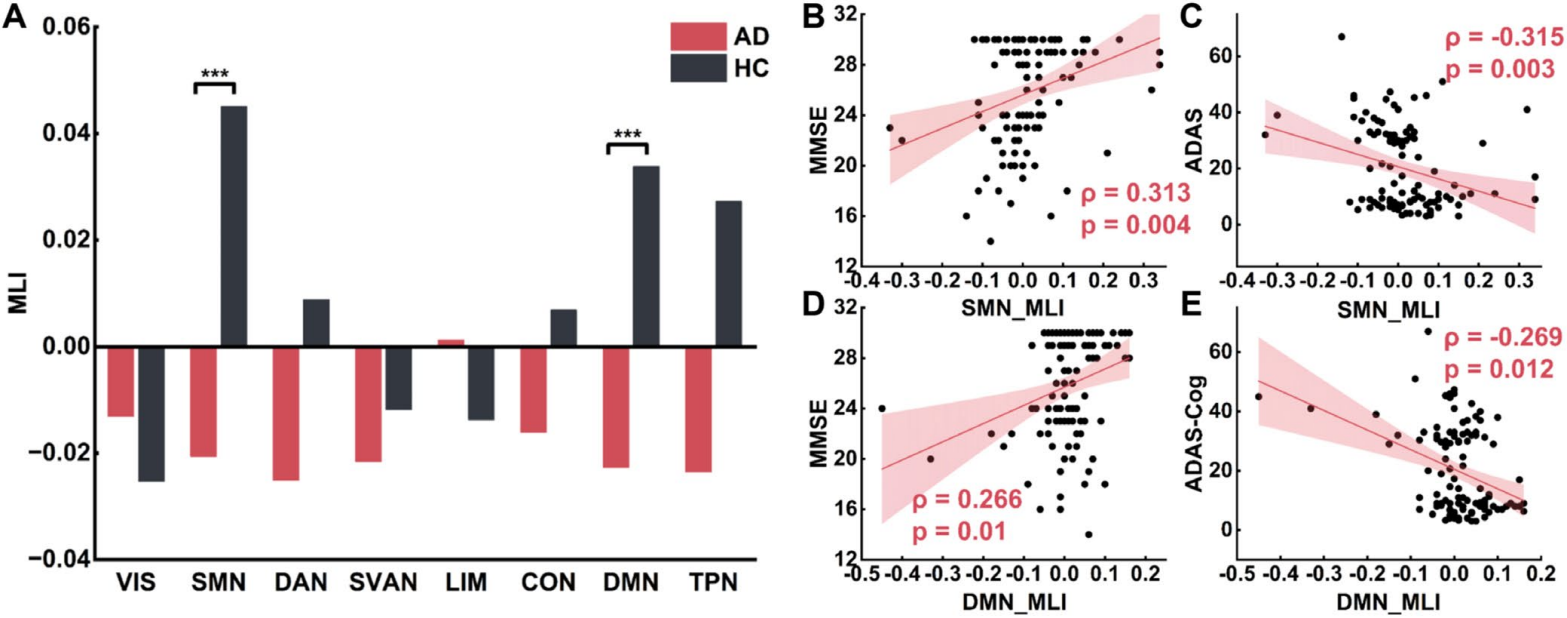
Rightward laterality of the SMN and DMN in AD and its association with cognitive impairment. (A) Comparison of MLI values between AD and HC groups across the eight functional networks. Bar plots depict the mean MLI value within each network for each group. *** indicates statistical significance at *p* < 0.001. (B) and (C) show the Spearman's correlations between the MLI and MMSE, and between the MLI and ADAS‐Cog, respectively, for the SMN network. (D) and (E) illustrate the corresponding correlation results for the DMN network. ADAS‐Cog, Alzheimer's Disease Assessment Scale; MMSE, Mini‐Mental State Examination.

Network‐level analyses revealed a global rightward shift of lateralization in AD. This was most evident in the SMN (AD: MLI = −0.0205 ± 0.098; HC: MLI = 0.0454 ± 0.091; *t* = −3.625, *p* < 0.001) and DMN (AD: MLI = −0.0218 ± 0.096; HC: MLI = 0.0341 ± 0.059; *t* = −3.754, *p* < 0.001), where the HC group showed leftward lateralization. The similar trends were observed in the DAN, CON, and TPN, although these differences did not reach statistical significance (Figure 2A).

Across all participants, lower MLI values in the SMN (*ρ* = 0.313, *p* = 0.004; Figure 2B) and DMN (*ρ* = 0.266, *p* = 0.01; Figure 2D) were associated with lower MMSE scores. Lower MLI values in the SMN (*ρ* = −0.315, *p* = 0.003; Figure 2C) and DMN (*ρ* = −0.269, *p* = 0.012; Figure 2E) were also associated with higher ADAS‐Cog scores across participants. ROI‐wise analyses showed similar trends in several regions (Table 2).

**TABLE 2:** Correlation of the MLI in the ROI with clinical features.

Network	ROI name	Correlation coefficient with MMSE	Correlation coefficient with ADAS
SMN	SomMotA	0.272*	−0.199
	SomMotB_Cent	0.283*	−0.235
	SomMotB_S2	0.203	−0.268*
DMN	DefaultA_PFCd	0.255*	−0.275*
	DefaultA_PCC	0.176	−0.304*
	DefaultC_Rsp	0.272*	−0.385*

*Note:* *indicates statistical significance at *p* < 0.05. SomMotA, somatomotor network; SomMotB_Cent, precentral gyrus; SomMotB_S2, secondary somatosensory cortex; DefaultA_PFCd, dorsal prefrontal cortex; DefaultA_PCC, posterior cingulate cortex; DefaultC_Rsp, retro splenial cortex.

### 3.3 Dynamic Lateralization in AD: Divergent Characteristics of LF and LR

**FIGURE 3:**
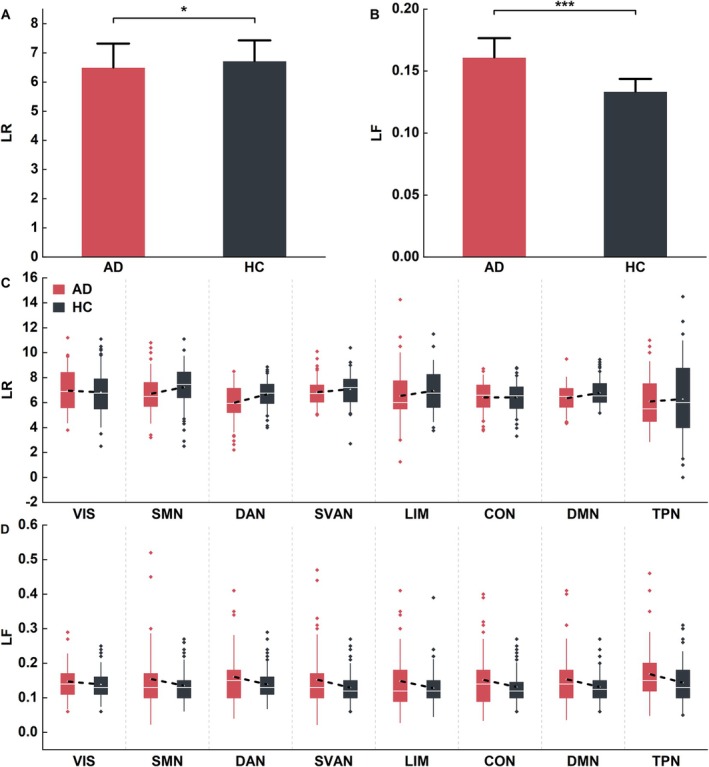
Differential dynamic laterality characteristics at the whole‐brain and subnetwork levels in participants with AD and HC. The comparison of mean LR (A) and LF (B) across 114 ROIs between the AD and HC groups, significant differences are denoted by * (*p* < 0.05) and *** (*p* < 0.001). Group differences in LR (C) and LF (D) for each network. The bars and error bars indicate mean and standard error, respectively, and the significant differences are indicated by ** (*p* < 0.01) and * (*p* < 0.05). LF, laterality fluctuation; LR, laterality reversal.

The dynamic lateralization characteristics LR and LF were compared between the AD and HC groups. AD showed opposing effects on these two metrics as shown in Figure 3. Compared with the HC group, participants with AD exhibited a significant decrease in LR (*t* = −2.183, *p* < 0.05; Figure 3A) and an increase in LF (*t* = 15.737, *p* < 0.001; Figure 3B). We observed global dynamic laterality changes and further analyzed each network separately. Both LR and LF showed global trends rather than network‐specific effects (Figure 3C–D).

### 3.4 LF–LR Interaction in AD: Disrupted Balance Depended on Lateralization Degree

**FIGURE 4:**
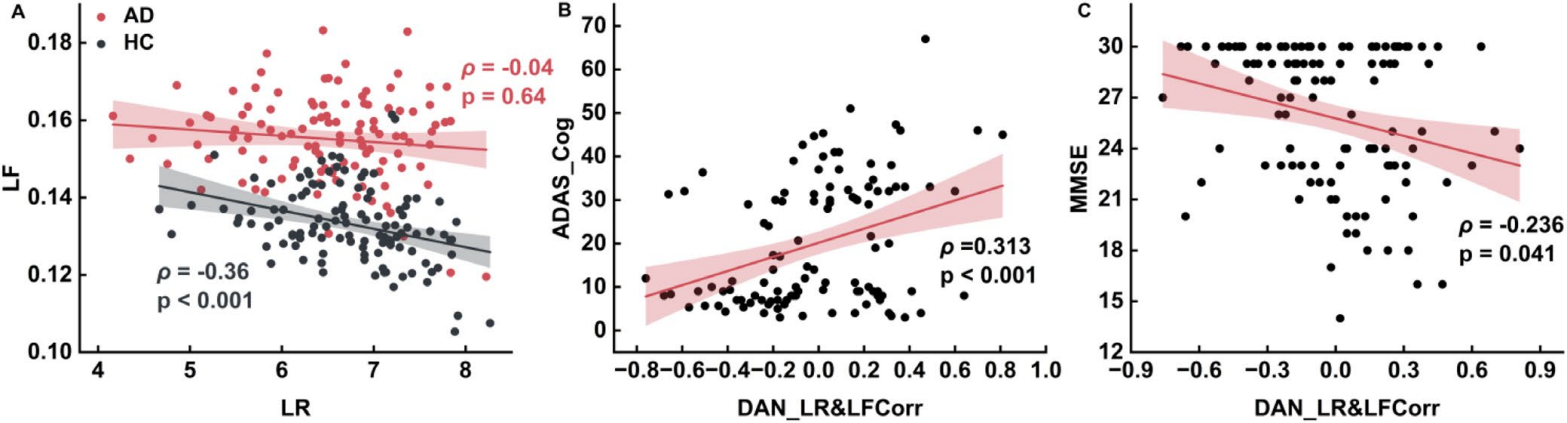
Loss of dynamic balance between the LR and LF in AD patients and its association with cognitive impairment. (A) At the group level, a significant correlation between LR and LF was detected in the HC group, while no such correlation was found in the AD group. At the individual level, LR and LF correlation (LR&LFCorr) calculations were calculated for each brain network. The results revealed a significant positive correlation between LR&LFCorr and ADAS‐Cog scores (B), and a significant negative correlation with MMSE scores (C) in the DAN. The Spearman correlation coefficient (*ρ*) and the *p* value calculated by the Spearman rank correlation test were used to assess the statistical significance of the correlation between the two indicators. LR&LFCorr, LR and LF correlation.

Next, we examined the Spearman correlation between LR and LF. At the group level, LR and LF were negatively correlated in the HC group (*ρ* = −0.36, *p* < 0.001; Figure 4A). In contrast, no such correlation was found in the AD group (*ρ* = −0.04, *p* = 0.64; Figure 4A). In the network‐based analysis of individual participants, significant associations were observed between the LR‐LF correlation coefficients and the clinical measures within the DAN. Specifically, within the DAN, the LR‐LF correlation coefficients exhibited a significant positive correlation with ADAS_Cog scores (*ρ* = 0.313, *p* < 0.001; Figure 4B). They also showed a significant negative correlation with MMSE scores (*ρ* = −0.236, *p* = 0.041; Figure 4C).

**FIGURE 5:**
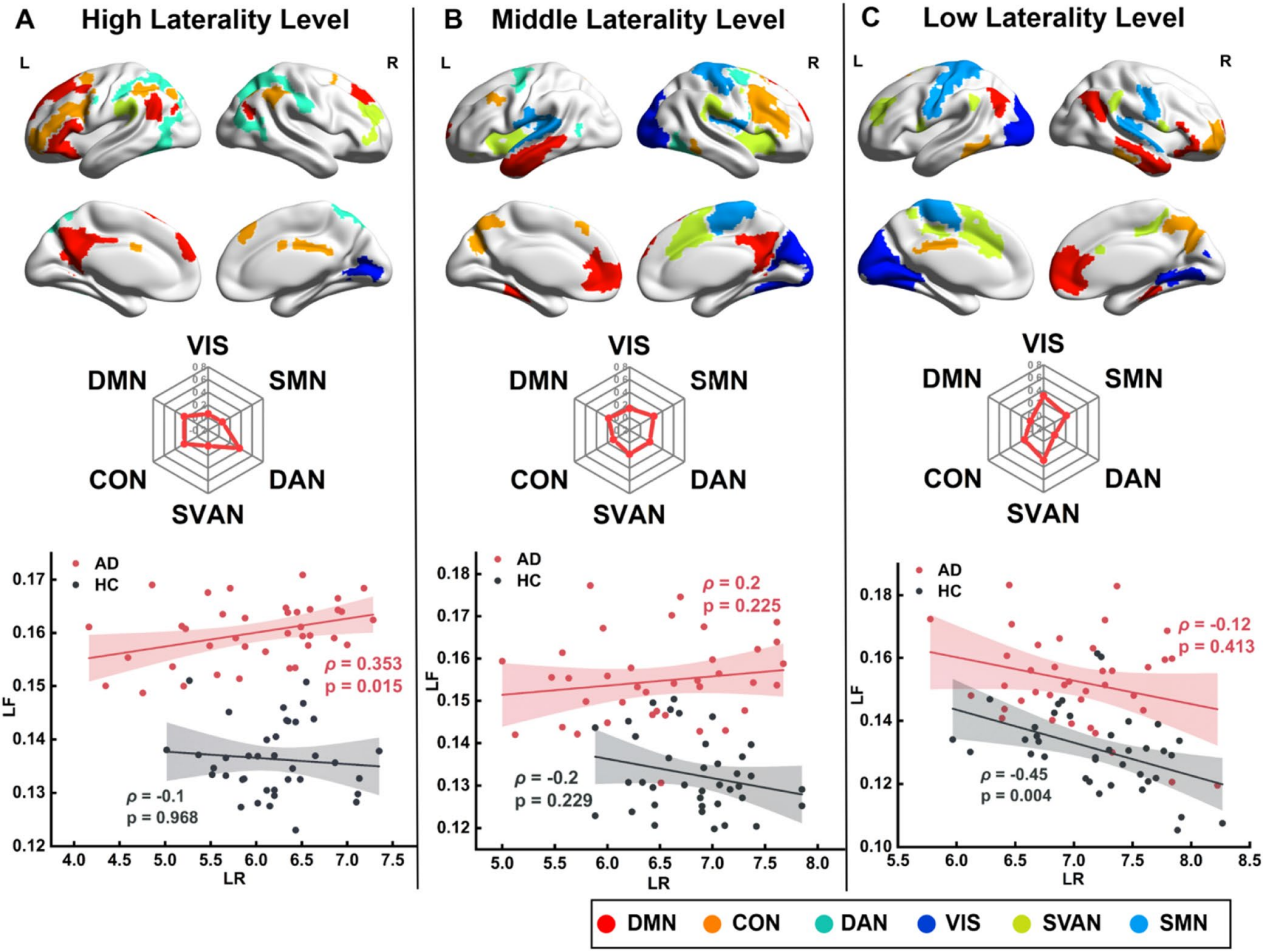
The degree of lateralization moderated the correlation between LR and LF. Spatial distributions of ROIs and the LR–LF correlation are shown at high (A), medium (B), and low (C) lateralization levels. The top row depicts the distribution of ROIs overlaid on the Montreal Neurological Institute‐152 brain template; the middle row illustrates the contributions of functional networks at each lateralization level; and the bottom row displays the correlation patterns of LR and LF in the AD and HC groups. The HC group presents a linear pattern, with increased opposing interactions between LR and LF as the lateralization degree declines. The AD group presented an inverted U‐shaped pattern, with an increased positive correlation between LR and LF observed at the high lateralization level. The Spearman correlation coefficient (*ρ*) and the *p* value calculated by the Spearman rank correlation test are used to assess the statistical significance of the correlation between the two indicators.

Having demonstrated the disruption of the opposing interaction between the LR and LF in the AD group, we further examined how the degree of lateralization influenced this alteration. As illustrated in Figure 5, the results revealed that the distribution of ROIs at different levels of lateralization varied in the weights of brain networks, showing a striking degree‐dependent association between LR and LF. ROIs with high lateralization were predominantly found in the DAN and CON (Figure 5A). ROIs with medium lateralization were mostly found in the SVAN (Figure 5B). ROIs with low lateralization were primarily located in the VIS and SVAN (Figure 5C). Correlation analysis at each level yielded the anticipated results. The disruption of dynamic balance between LR and LF in the AD group was mainly driven by regions with high lateralization. At the high lateralization level, the AD group showed a significant positive correlation between LR and LF (*ρ* = 0.353, *p* = 0.015; Figure 5A), whereas no such correlation was found in the HC group (*ρ* = −0.1, *p* = 0.968; Figure 5A). At the medium lateralization level, no significant correlation between LR and LF was found in either group. At the low lateralization level, a negative correlation between LR and LF was found only in the HC group (*ρ* = −0.45, *p* = 0.004; Figure 5C). That is, the HC group exhibited a monotonic pattern in the interaction between LR and LF, with an increasing degree of lateralization, whereas the AD group displayed an inverted U‐shaped pattern. Additionally, the results based on the Schaefer parcellation demonstrated a widespread weakening, but not a complete loss, of the negative LR‐LF correlation in AD (see Supporting Information). Specifically, the strength of this correlation followed a continuous gradient, being most severely disrupted in highly lateralized regions while partially retained in weakly lateralized ones. This pattern of graded impairment suggests that the anomalous positive correlation observed with the Yeo atlas may reflect a distinct, pathologically driven reorganization within specific higher‐order networks. Together, these findings highlight the subtle influence of lateralization degree on the dynamic balance between the LR and LF, particularly in the context of AD.

## 4 Discussion

This study aimed to explore the dynamic changes in hemispheric lateralization among participants with AD. Our findings revealed a significant increase in lateralization across both hemispheres in the AD group. This effect was particularly pronounced as a rightward shift in the SMN and DMN. Further dynamic analysis demonstrated that these rightward shifts co‐occur with decreased LR and increased LF. This pattern reflects concurrent disruptions in both intra‐hemispheric processing and inter‐hemispheric communication. Crucially, these temporal dynamics are undetectable with static measures, underscoring the need for time‐resolved assessment. We introduce the LF–LR interaction pattern as a novel AD biomarker, showing its lateralization‐dependent and network‐specific disruption captures how impaired dynamic lateralization contributes to cognitive decline. Importantly, these abnormal lateralization metrics were associated with diminished MMSE scores and elevated ADAS‐Cog scores. Our findings highlight the alterations in intra‐ and interhemispheric information communication, suggesting fundamental changes in brain network organization associated with AD.

Both anatomical and functional imaging studies have convincingly demonstrated brain lateralization (Gotts et al. 2013). Healthy elderly subjects typically exhibit leftward lateralization, which is likely associated with language dominance (Smith et al. 1996). In contrast, brain injuries or neurological disorders often lead to altered hemispheric lateralization, which affects cognitive abilities (Gotts et al. 2013). Specifically, in our AD cohort, the normal leftward lateralization observed in most subnetworks of healthy elderly individuals was diminished. Instead, we noted rightward lateralization in the SMN and DMN in this cohort. The shift in lateralization may be due to AD‐specific structural lesions, as reported in the literature. Previous studies have shown that gray matter loss in AD first appears in the entorhinal and temporal–parietal cortices, and later extends to the frontal and sensorimotor regions as the disease advances (Thompson et al. 2003, 1998). The asymmetrical patterns of neurodegeneration indicate that the left hemisphere is more vulnerable than the right (Thompson et al. 1998; Roe et al. 2021; Li et al. 2009). We therefore speculate that the decreased brain activation observed in the left hemisphere of AD patients could be linked to the loss of left‐lateralized neurons along the trajectory of gray matter loss (Bozzali et al. 2006). On the other hand, the degeneration of the commissural WM bundles connecting the two hemispheres in AD patients may contribute to the abnormal rightward shift in lateralization (Qiu et al. 2016). This degeneration has been reported to weaken functional connectivity between homologous brain regions across hemispheres, thereby enhancing the functional lateralization of interhemispheric interactions (Jin et al. 2024). This finding aligns with our observation of increased lateralization within both hemispheres. Notably, Pariente et al. found that cognitive performance was correlated with increased right hemisphere activation during semantic and episodic tasks, suggesting that right lateralization is related to functional compensation (Pariente et al. 2005; Grady et al. 2003). However, our resting‐state study revealed an opposing correlation between right lateralization and cognitive function. This discrepancy may arise from differences in task demands and cognitive processes involved, as well as variations in neural network engagement during resting states compared to active cognitive tasks (Hearne et al. 2017). More importantly, the loss of normal leftward lateralization and the emergence of abnormal rightward lateralization in AD patients impede compensatory mechanisms and disrupt interhemispheric interactions. These alterations ultimately reduce the brain's working efficiency (Liu et al. 2018). Overall, while our findings highlight significant alterations in brain lateralization in AD, further research is needed to explore the specific mechanisms underlying these changes and their implications for cognitive function.

Importantly, we observed abnormal dynamic laterality characteristics in AD patients. In comparison to the HC group, the AD group exhibited a decrease in global LR and an increase in global LF. Based on the calculation principles of LR and LF, we speculate that these changes have different origins. The reduced LR likely reflects fewer hemispheric reversals, while the increased LF indicates more diverse fluctuations in inter‐network connectivity. Consistent with the observed decrease in hemispheric reversals, loss of intra‐network connectivity has been reported in AD. This loss affects both intra‐ and inter‐hemispheric connections across large‐scale networks, including the DMN, DAN, CON, and SVAN (Liu et al. 2018; Brier et al. 2012). To some extent, functionally linked resting‐state networks reflect underlying structural connectivity (Honey et al. 2009; Greicius et al. 2009) and vice versa. Abnormalities in inter‐hemispheric WM pathways could influence functional connectivity patterns, including the information exchange between hemispheres (Filippi 2011; Wang et al. 2015). The corpus callosum, the largest commissural WM bundle in the human brain, primarily connects homologous regions of the cortex and serves as the main conduit for information transfer between the two cerebral hemispheres. Previous studies have shown that AD patients exhibit reduced midsagittal area, thickness, and volume of the corpus callosum compared with controls (Di Paola et al. 2010; Wang et al. 2005). This degeneration further disrupts interhemispheric homotopic information transfer (Qiu et al. 2016). Moreover, previous studies have explored how corpus callosum degeneration correlates topographically with inter‐hemispheric homotopic functional connectivity in AD patients. Specifically, disruption of inter‐hemispheric homotopic functional connectivity in AD mainly occurs in the temporal–parietal regions, including the precuneus, posterior cingulate cortex, and hippocampus, which are key areas of the DMN (Teipel et al. 2010; Cavanna and Trimble 2006). In addition, it has been reported that AD patients exhibit increased dynamic functional connectivity variability in widespread regions, including the DMN and frontoparietal network (Niu et al. 2019), which aligns with the observed increase in LF in the AD group. The LF is fundamentally linked to intra‐ hemispheric information processing and is positively associated with cognition (Wu et al. 2022). In this context, we argue that the observed increase in LF can be interpreted as a compensatory mechanism for inter‐hemispheric communication. As intra‐hemispheric processing becomes less efficient due to structural and functional decline in AD, the brain may compensate by increasing the LF. This enhancement may improve the flexibility of interhemispheric communication. This dynamic flexibility could allow the brain to recruit alternative neural resources in both hemispheres, particularly in response to functional loss in some regions. Previous research has demonstrated that AD patients recruit more widespread or alternative brain regions as compensation for functional losses (Scheller et al. 2014). While such adaptations may be beneficial in the early stages, they may also reflect a cost: the increase in LF could signify a trade‐off between global integration and local specialization. This trade‐off could enhance the efficiency of information spreading across the whole brain, while simultaneously resulting in a loss of efficacy in information dissemination at the finer scales of vertices and their neighbors. Interestingly, although no specific network demonstrated a significant increase in LF in AD patients, all networks exhibited upward trends. This suggests that the shift may not be isolated to individual systems but represents a whole‐brain phenomenon. Similar to the observation that brain‐wide integration increases as the disease progresses or during task demands in aging (Fathian et al. 2022; Crowell et al. 2020), AD appears to drive functional networks toward a more random architecture. Consequently, the increase in LF likely reflects a complex, dynamic reorganization process where compensatory adaptations are ultimately constrained by the underlying network architecture and disease progression.

From the perspective of neural network dynamics, LR and LF are considered to represent intra‐hemispheric and inter‐hemispheric interactions and cooperation, respectively (Wu et al. 2022). Certain cognitive abilities require stronger inter‐hemispheric interaction than intra‐hemispheric processing to maintain stable function, as seen in networks such as the DMN and DAN (Gao et al. 2023). In contrast, other cognitive abilities rely primarily on one hemisphere. For example, the right hemisphere supports attention and perception, whereas the left hemisphere supports language processing. This functional specialization leads to increased local intra‐hemispheric circuitry and reduced inter‐hemispheric communication (Gazzaniga 2000). Consequently, cognitive processes are associated with a dynamic balance between inter‐hemispheric information exchange and intra‐hemispheric information processing (Wu et al. 2022), as observed in the HC group. In contrast, the loss of the negative association between LR and LF in AD patients suggests a disrupted dynamic balance between these two processes. To explore this phenomenon more deeply, we investigated the effects of different levels of laterality on the dynamic balance. Our multi‐atlas analysis indicates that AD impairs dynamic hemispheric interactions. The disruption follows a hierarchical pattern, with severity forming a continuous gradient according to the degree of regional lateralization. This graded impairment is most pronounced in highly lateralized regions that support higher order cognition. It aligns with established neuropathological models of AD, in which pathology first affects higher order association cortices and then progressively involves other regions (Braak and Braak 1991). The particular vulnerability of highly lateralized networks appears related to their dependence on intact interhemispheric connectivity. Previous research has demonstrated that corpus callosum degeneration correlates topographically with disrupted interhemispheric homotopic functional connectivity in temporal–parietal regions of AD patients (Teipel et al. 2010; Cavanna and Trimble 2006). Our observation that networks like the DMN and DAN show the most severe balance disruption supports this connection, suggesting that WM pathway deterioration compromises highly lateralized networks' ability to maintain their specialized functions. The aberrant correlation patterns observed in high‐lateralization regions may represent attempted compensatory reorganization or pathological network alterations following normal interhemispheric communication breakdown.

This vulnerability pattern aligns with the proposal that regions with lower laterality levels demonstrate greater integration flexibility in both intra‐ and inter‐hemispheric interactions, while regions with higher levels of laterality are more functionally specialized and relatively segregated (Wu et al. 2022). Consequently, we speculate that disruptions in this balance are more pronounced in regions characterized by higher lateralization, which are inherently more specialized, compared to areas with lower levels of biased laterality that exhibit greater adaptability. Our findings support this hypothesis, revealing that the alterations in the relationship between LR and LF in AD patients were predominantly observed in regions with higher levels of lateralization. Furthermore, a weight analysis of the networks indicated that higher‐order cortical networks, such as the DAN and the DMN, exhibited greater weights in regions with higher levels of laterality. In contrast, regions with lower levels of laterality showed relatively higher weights associated with primary cortical networks, including the VIS (Zheng et al. 2024). Taken together, our findings highlight the critical role of lateralization in the dynamic balance observed in AD patients. We provide additional evidence of disturbances in the lateralized structure of the brain, suggesting that regions with higher lateralization may be more vulnerable to disruptions, whereas those with lower lateralization retain a degree of flexibility. These changes may be linked to underlying cognitive deficits in AD, particularly in attention and cognitive control (Corbetta et al. 2008; Devanand et al. 2008; Verghese et al. 2007; Vipin et al. 2018; Zhu et al. 2016). This insight could inform future research and therapeutic strategies aimed at addressing cognitive impairments in AD.

## 5 Limitations

There are several limitations to this study that need to be considered. First, handedness was included as a covariate in our statistical models to reduce its potential influence. However, a key limitation is that most participants were right‐handed. Since handedness is linked to brain lateralization (Gazzaniga 2000), the generalizability of our findings to left‐handed populations is unclear. Future studies should include a balanced sample of left‐ and right‐handed participants to validate and extend our conclusions. Therefore, the findings of this study should be further validated in future studies involving left‐handed participants to ensure their generalizability. Additionally, our study utilized resting‐state data; we were unable to relate the results to specific cognitive processes or abilities. Future research should examine dynamic lateralization differences between AD patients and HC during specific cognitive tasks. This approach would provide a more comprehensive understanding of these dynamics. Finally, neurodegenerative diseases are characterized by progressive cognitive decline as the disease advances (Gonzales et al. 2022; Sheffield et al. 2018). This study employs a cross‐sectional framework to investigate the dynamic lateralization differences between patients with AD and HC. However, due to the lack of longitudinal data tracking the participants, we are unable to comprehensively explore the evolution of brain lateralization features during the cognitive decline process. Further studies should focus on the dynamic changes in brain lateralization from MCI to severe cognitive impairment, ultimately progressing to dementia. Such studies could provide crucial insights into the relationship between cognitive decline and alterations in brain structure and function. They would contribute to the development of a more robust scientific foundation for early diagnosis and targeted interventions.

## 6 Conclusions

In summary, our study revealed alterations in the dynamic features of brain lateralization in AD patients examined from both the whole brain and subnetwork perspectives. We found increased hemispheric lateralization and abnormal right‐hemisphere dominance in the SMN and DMN of AD patients. These changes were associated with cognitive impairment. Additionally, we observed divergent dynamic laterality characteristics in LR and LF in AD patients. This suggests disruptions in inter‐hemispheric functional connectivity. It may also reflect compensatory mechanisms for inter‐hemispheric communication. Notably, the negative association between the LR and LF observed in HC was absent in AD patients. To our knowledge, this is the first study to provide direct evidence that the dynamic balance between intra‐ and inter‐hemispheric information interactions is disrupted in AD. Importantly, this imbalance was found to be lateralization dependent, with highly lateralized networks being particularly vulnerable. These findings underscore the potential of dynamic lateralization metrics as biomarkers for disease monitoring and intervention assessment. Future studies are needed to establish their clinical value.

## Author Contributions

J.W., Y.L., and Y.Y. designed the study and also contributed to the data processing, statistical analysis interpretation of the results, and writing of the manuscript. S.L., X.C., P.X., X.L., and Y.G. contributed to the critical revision of the manuscript. All authors read and approved the final manuscript.

## Ethics Statement

All enrolled participants or authorized representatives provided informed consent, approved by ADNI center's respective Institutional Review Boards, which were in accordance with the ethical standards of the institutional and/or national research committee and with the Declaration of Helsinki.

## Consent

The authors have nothing to report.

## Conflicts of Interest

The authors declare no conflicts of interest.

## Supporting information


**Data S1:** hbm70411‐sup‐0001‐Supinfo.docx.

## Data Availability

All data used in this manuscript is publicly available from the ADNI database (https://adni.loni.usc.edu) upon registration and compliance with the data use agreement. The data that support the findings of this study are available on reasonable request from the corresponding author.
