# Supplementary material for: Lateralization Disruption and Dynamic Balance Alterations in Alzheimer's Disease: Impacts on Hemispheric Interaction and Cognitive Performance
Source: Hum Brain Mapp. 2025 Nov 14;46(16):e70411. doi: 10.1002/hbm.70411 (PMC12616499; doi:10.1002/hbm.70411)
Supplement: Supplementary file 1 — Data S1: hbm70411‐sup‐0001‐Supinfo.docx. [file HBM-46-e70411-s001.docx]

**Supplementary Methods and Results**

### Sensitivity Analysis of Dynamic Lateralization Measures: Effects of Sliding Window Length and Step Size

To evaluate the robustness of our dynamic laterality metrics to parameter selection, we performed sensitivity analyses using different sliding window lengths (15 TR, 25 TR, and 30 TR) and step sizes (2 TR and 3 TR). For each region of interest (ROI), we recomputed the three dynamic indices, including mean laterality index (MLI), laterality fluctuation (LF), and laterality reversal (LR), under each alternative window setting. We then computed Pearson correlation coefficients between ROI-wise values derived from the primary parameter set (20-TR window, 1-TR step) and those from each alternative setting, separately for the Alzheimer’s disease (AD) and healthy control (HC) groups.

Overall, most indices showed high correlations (r > 0.7; Figures S1–S2) across parameter settings. Notably, LR computed with a 30-TR window exhibited somewhat lower—though still consistent—correlations (r = 0.5~0.8; Figure S1). After false discovery rate (FDR) correction (q < 0.05), none of the correlations differed significantly, confirming that our findings are robust to parameter variation.

## Analysis of Head Motion Effects on Dynamic Laterality Metrics

We further verified that the observed dynamic laterality measures were not confounded by head motion. Spearman correlations were calculated between mean framewise displacement (FD) and each dynamic laterality metric (MLI, LR, LF) across eight functional networks. After false discovery rate (FDR) correction (q < 0.05), no significant correlations were found (all p > 0.05), demonstrating that residual motion did not influence the results (Table S1).

### Effects of Global Signal Regression on Dynamic Laterality Metrics

Because global signal regression (GSR) can distort interhemispheric relationships that are critical for laterality computation, it was not applied in our main analysis. To empirically verify that the global signal did not significantly influence the dynamic laterality index (DLI), we performed a regression analysis in which each ROI’s DLI time series was regressed against the global signal. The resulting t-values for the regression coefficients were then tested against zero. After FDR correction (q < 0.05), no ROI showed a significant association, and the t-values remained consistently near zero across all ROIs (Figure S3). These results confirm that the global signal does not systematically affect the dynamic laterality measures.

### Brain Parcellation derived from the Yeo 17-Network Atlas

For all analyses in this study, the cerebral cortex was parcellated into 114 cortical regions of interest (ROIs), equally divided between the two hemispheres, based on the functional atlas developed by Yeo et al. [1]. This atlas was derived from large-scale intrinsic functional connectivity analyses of 1,000 healthy individuals and has been widely applied in resting-state fMRI studies to characterize large-scale network organization. Each ROI was assigned to one of 17 intrinsic functional networks consistent with the original Yeo17 network definition (Table S2). These networks were further grouped into eight major functional systems for network-level analysis: the Visual (VIS), Somatomotor (SMN), Dorsal Attention (DAN), Ventral Attention/Salience (SVAN), Limbic (LIM), Control (CON), Default Mode (DMN), and Temporoparietal (TPN) networks. This parcellation scheme ensures a balanced and symmetric division of cortical regions across hemispheres, enabling consistent mapping of functional networks between the left and right hemispheres. It has been shown to provide high reproducibility and robustness in analyses of dynamic functional connectivity and brain lateralization.

## Validation analyses using the Schaefer 400-ROI Parcellation

To examine the robustness of the findings, we repeated our analysis using Schaefer parcellation with 400 ROIs [2]. It is considered a spatially refined version of the Yeo network template. The network-wise mean lateralization indices (MLIs) were first calculated for each hemisphere, yielding eight bilateral MLI pairs per cohort (Fig. S4). Consistent with the results obtained using the 114-ROI template, participants with AD showed significantly greater rightward lateralization within the right-hemispheric SMN (t = −4.04, p = 0.003), DMN (t = −2.41, p = 0.03), and TPN (t = −2.68, p = 0.02) (Fig. S4D). Some differences, however, were also observed. Compared with the HC group, the AD group exhibited enhanced lateralization in both hemispheres across all networks except the SMN, LIM, DMN, and TPN. Among these, the SMN, DMN, and TPN showed increased rightward lateralization confined to the right hemisphere.

Hemispheric-level analyses across the eight networks further supported these findings. Relative to HCs, individuals with AD demonstrated an overall rightward shift, particularly within the SMN (t = −3.75, p < 0.001) and DMN (t = −3.27, p = 0.003), whereas the HC group displayed leftward lateralization. Similar rightward tendencies were observed in the DAN, CON, and TPN, although these differences did not reach statistical significance (Fig. S5A). Across all participants, lower MLI values in the SMN (ρ = 0.298, p = 0.007; Fig. S5B) and DMN (ρ = 0.253, p = 0.02; Fig. S5D) correlated with lower MMSE scores. Conversely, lower MLI values in the SMN (ρ = −0.359, p = 0.001; Fig. S5C) and DMN (ρ = −0.223, p = 0.04; Fig. S5E) were associated with higher ADAS-Cog scores.

Dynamic lateralization characteristics (LR and LF) were then compared between groups (Fig. S6). As in the 114-ROI analysis, AD exerted opposing effects on these two measures. Compared with HCs, individuals with AD exhibited significantly decreased LR (t = −3.67, p < 0.001; Fig. S6A) and increased LF (t = 26.05, p < 0.001; Fig. S6B). These global alterations were observed across the entire brain and were not driven by any specific network. Similar to the 114-ROI results, LF increased across all networks, whereas a distinct finding under the 400-ROI template was that LR decreased across all networks except the CON and TPN (Fig. S6C–D).

The relationship between LR and LF was further examined using Spearman correlation. At the group level, a significant negative correlation between the group-averaged LR and LF was found in both the AD and HC groups (AD: ρ = −0.217, p < 0.001; HC: ρ = −0.318, p < 0.001; Fig. S7A). Notably, the correlation tended to be weaker in the AD group (Spearman’s r = −0.217 vs. −0.318; Fisher’s r-to-z = −1.53, p = 0.06). Consistent with the 114-ROI findings, network-level analyses revealed that LR–LF correlation coefficients within the DAN were significantly associated with clinical measures—positively with ADAS-Cog scores (ρ = 0.229, p = 0.03; Fig. S7B) and negatively with MMSE scores (ρ = −0.253, p = 0.03; Fig. S7C).

Finally, a stratified analysis was performed based on intrinsic lateralization strength. The distribution of ROIs across different lateralization levels varied in network composition (Fig. S8). Highly lateralized ROIs were mainly located within the DMN and CON (Fig. S8A), those with medium lateralization were concentrated in the DMN and SMN (Fig. S8B), and low-lateralization ROIs were primarily distributed in the VIS and SMN (Fig. S8C). In the AD group, the negative LF–LR correlation reached only marginal significance in low-lateralization regions (Fig. S8C) and was absent in regions with medium or high lateralization (Fig. S8A–B). In contrast, the HC group exhibited stable negative LF–LR correlations across both medium- and low-lateralization regions (Fig. S8B–C).

Combined evidence from two brain parcellations indicates that Alzheimer's disease is associated with global rightward shifts in static hemispheric lateralization. Dynamic measures show opposing changes, with reduced LR and elevated LF. These alterations follow a lateralization-strength–dependent pattern. The effect is most pronounced in higher-order cognitive networks. Importantly, these changes are closely linked to cognitive decline.

# Supplementary figures

**
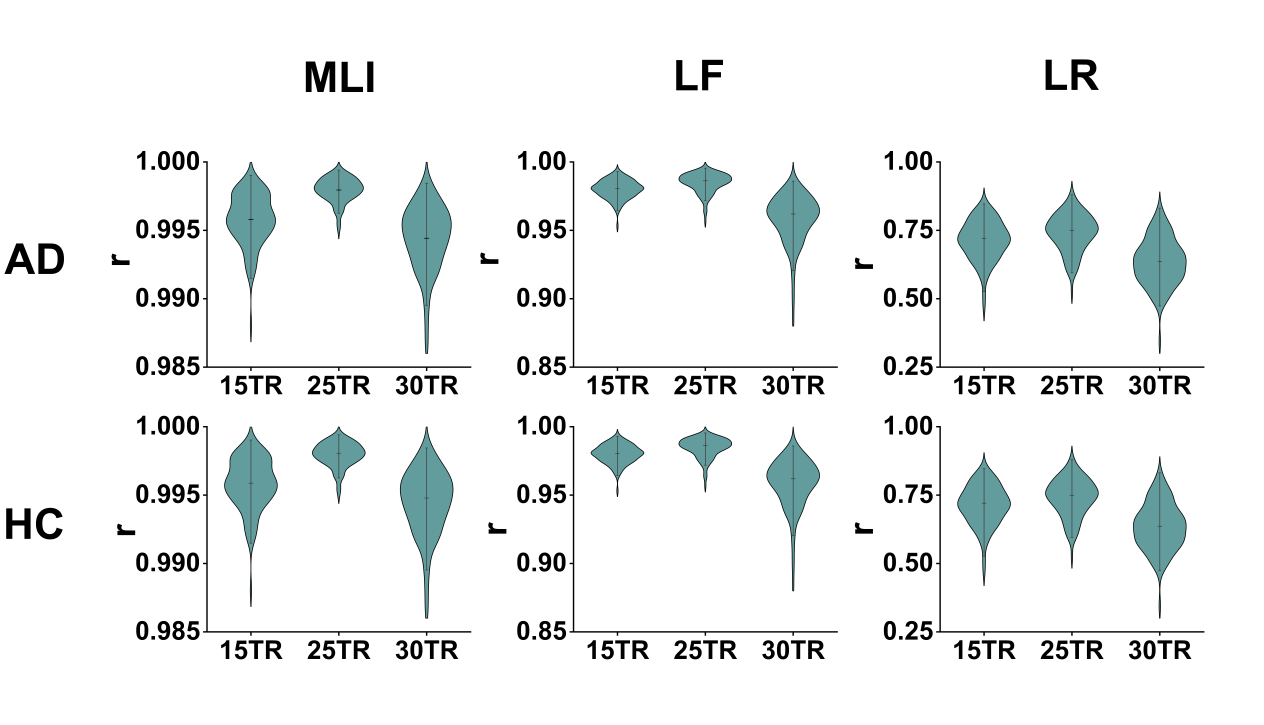
**

**Figure S1. Correlation of features derived from a 20-TR window with those obtained using alternative window lengths in the AD and HC groups.**


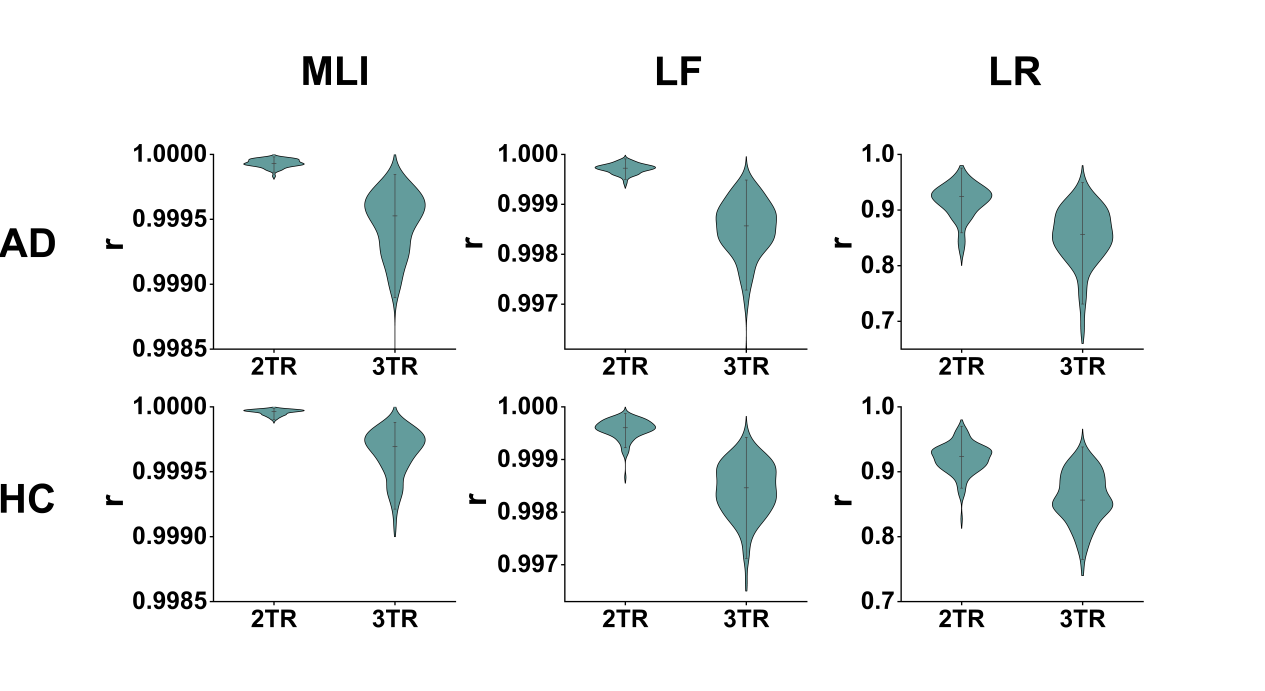


**Figure S2. Correlation of features derived with a 1-TR step size and those obtained using alternative step sizes in the AD and HC groups.**

**
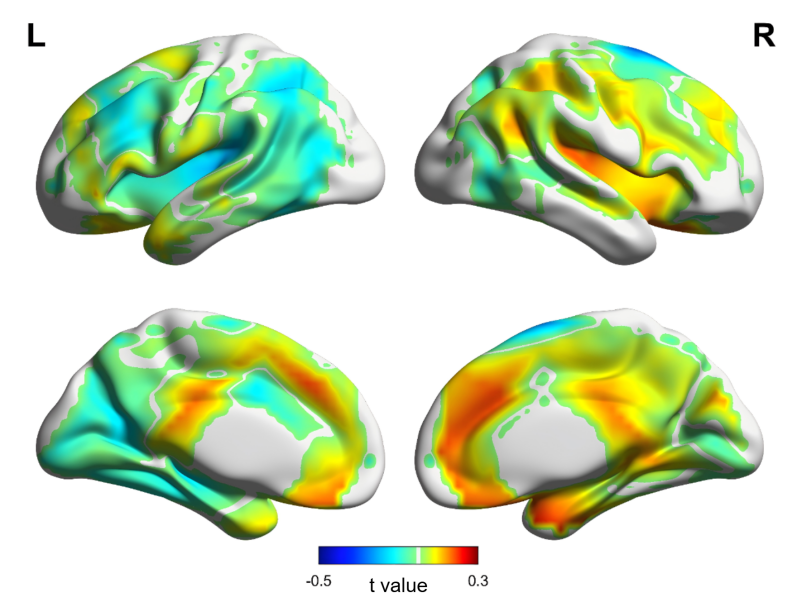
**

**Figure S3. Absence of a significant association between the global signal and dynamic laterality indices. The brain map shows the t-values from regressing each ROI’s dynamic laterality index (DLI) time series against the global signal.**

**
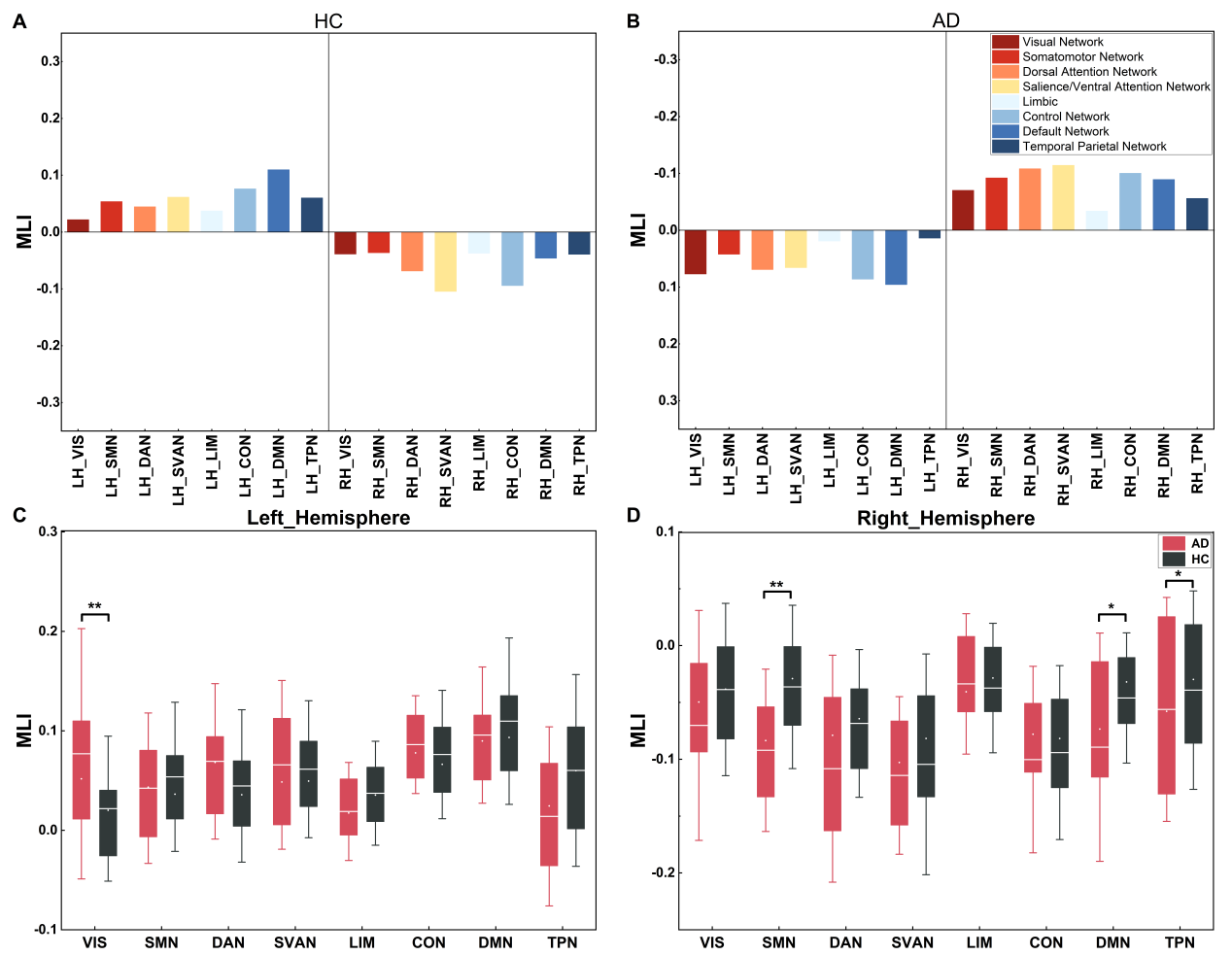
**

**Figure S4. The spatial distribution of the dynamic laterality in the larger-scale brain networks.** The mean MLI values for the 16 subnetworks in the left and right hemispheres of the HC group (A) and the AD group (B). The disparities in MLI values observed between the eight networks in the left (C) and right (D) hemispheres of subjects in the AD and HC groups. A total of 114 ROIs were divided into 16 subnetworks based on their location in the left or right hemisphere. The MLI values of the ROIs in each region were then averaged to obtain the MLI value for each of the 16 subregions. A positive MLI of a region indicates stronger interaction with the left hemisphere (leftward laterality), whereas a negative MLI indicates rightward laterality. HC, healthy control; AD, Alzheimer‘s disease; MLI, mean laterality index; LH, left hemisphere; RH, right hemisphere; VIS, visual network; SMN, somatomotor network; DAN, dorsal attention network; SVAN, salience/ventral attention network; LIM, limbic; CON, control network; DMN, default mode network; TPN, temporal parietal network. ** indicates significance of p < 0.01. * indicates significance of p < 0.05.

**
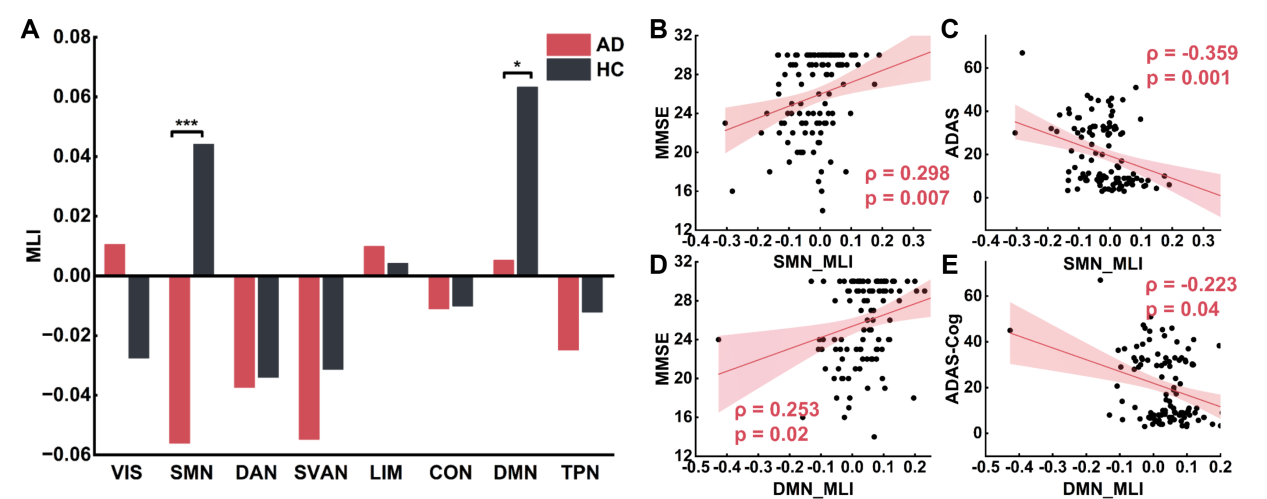
**

**Figure S5. Rightward laterality of the SMN and DMN in AD and its association with cognitive impairment.** (A) Comparison of MLI values between AD and HC groups across the eight functional networks. Bar plots depict the mean MLI value within each network for each group. (B) and (C) show the Spearman’s correlations between the MLI and MMSE, and between the MLI and ADAS-Cog, respectively, for the SMN network, respectively. (D) and (E) illustrate the corresponding correlation results for the DMN network. MMSE, Mini-Mental State Examination; ADAS-Cog, Alzheimer’s Disease Assessment Scale. *** indicates statistical significance at p < 0.001. ** indicates statistical significance at p < 0.01.

**
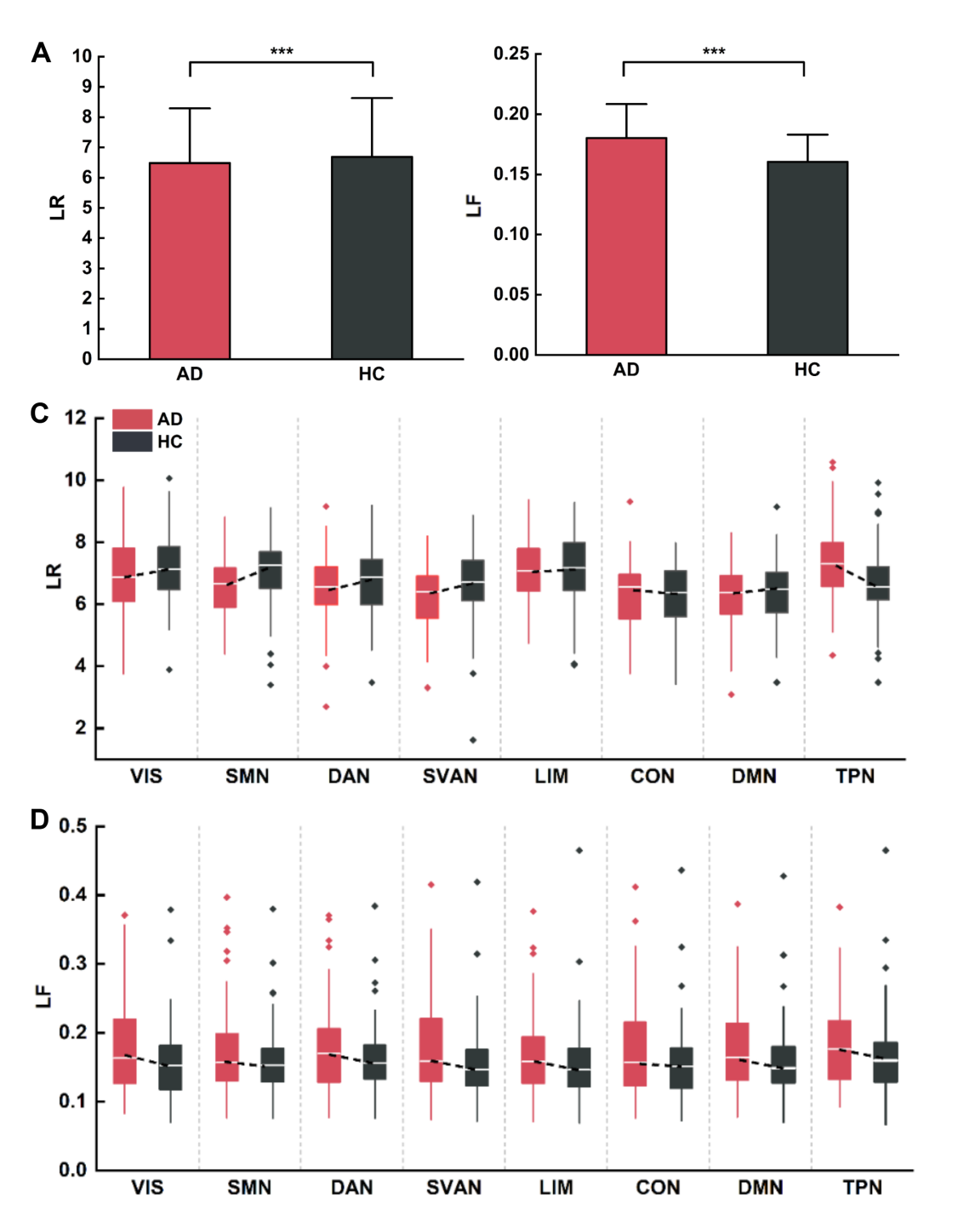
**

**Figure S6. Differential dynamic laterality characteristics at the whole-brain and subnetwork levels in participants with AD and HC.** The comparison of mean LR (A) and LF (B) across 114 ROIs between the AD and HC groups. Group differences in LR (C) and LF (D) for each network. The bars and error bars indicate mean and standard error respectively. LR, laterality reversal; LF, laterality fluctuation. *** indicates statistical significance at p < 0.001.

**
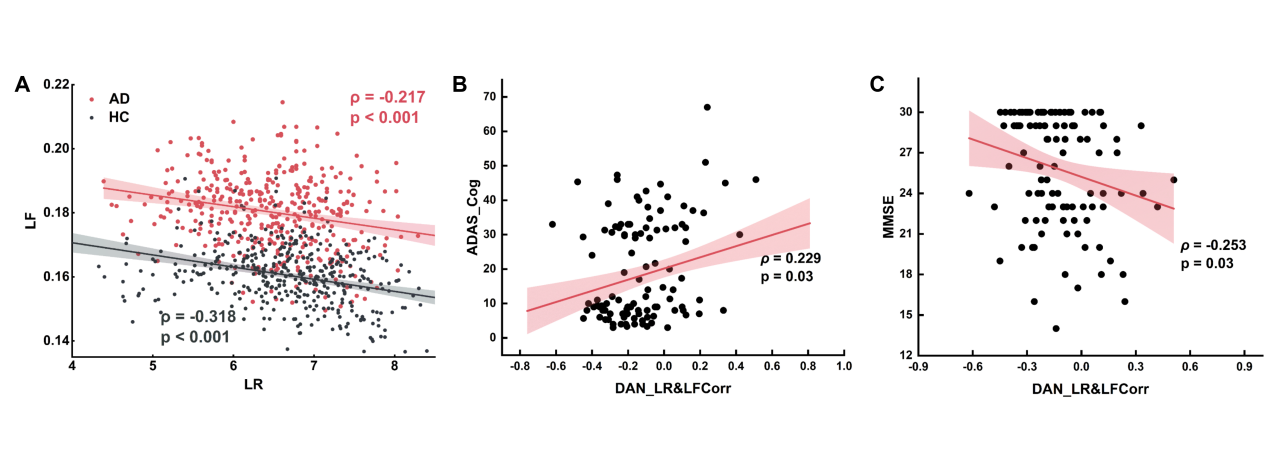
**

**Figure S7. Weaken of dynamic balance between the LR and LF in AD patients and its association with cognitive impairment.** (A) At the group level, a significant correlation between LR and LF was detected in the HC group, while no such correlation was found in the AD group. At the individual level, LR and LF correlation (LR&LFCorr) calculations were calculated for each brain network. The results revealed a significant positive correlation between LR&LFCorr and ADAS-Cog scores (B), and a significant negative correlation with MMSE scores (C) in the DAN. The Spearman correlation coefficient (*ρ*) and the p-value calculated by the Spearman rank correlation test were used to assess the statistical significance of the correlation between the two indicators. LR&LFCorr, LR and LF correlation.

**
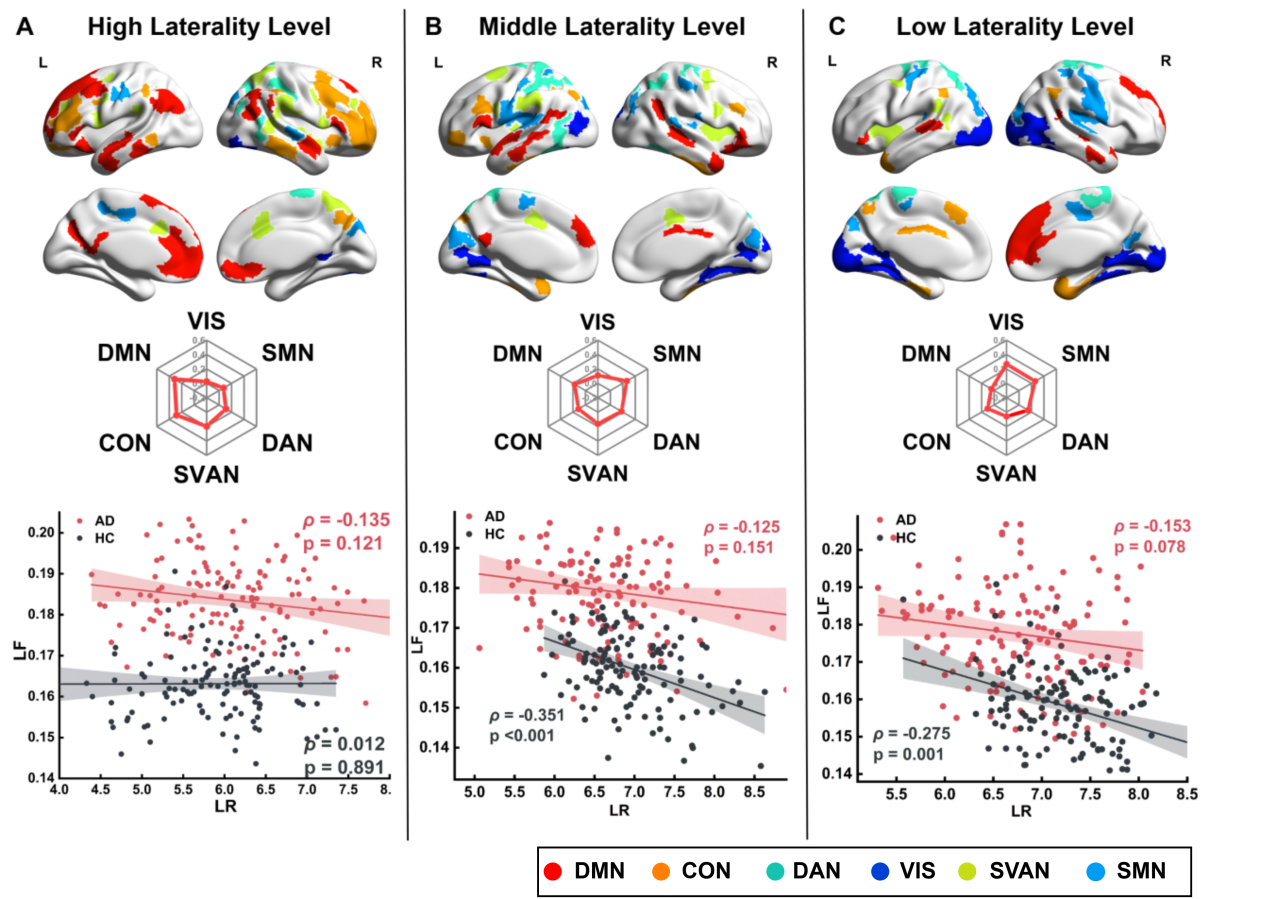
**

**Figure S8. The degree of lateralization moderated the correlation between LR and LF.** Spatial distributions of ROIs and the LR–LF correlation are shown at high (A), medium (B), and low (C) lateralization levels. The top row depicts the distribution of ROIs overlaid on the Montreal Neurological Institute-152 brain template; the middle row illustrates the contributions of functional networks at each lateralization level; and the bottom row displays the correlation patterns of LR and LF in the AD and HC groups. The HC group presents a linear pattern, with increased opposing interactions between LR and LF as the lateralization degree declines. The AD group presented an inverted U-shaped pattern, with an increased positive correlation between LR and LF observed at the high lateralization level. The Spearman correlation coefficient (*ρ*) and the p-value calculated by the Spearman rank correlation test are used to assess the statistical significance of the correlation between the two indicators.

# Supplementary tables

**Table S1. Correlations between mean FD and dynamic laterality measures across all functional networks.**

| **Network** | **Mean FD vs. MLI** | **Mean FD vs. LR** | **Mean FD vs. LF** |
| --- | --- | --- | --- |
| **VIS** | r = -0.13, p = 0.30 | r = -0.05, p = 0.40 | r = 0.05, p = 0.40 |
| **SMN** | r = -0.19, p = 0.19 | r = 0.16, p = 0.23 | r = 0.01, p =0.48 |
| **DAN** | r = -0.21, p = 0.19 | r = -0.10, p = 0.32 | r = 0.07, p = 0.35 |
| **SVAN** | r = -0.20, p = 0.19 | r = -0.07, p = 0.35 | r = 0.03, p = 0.42 |
| **LIM** | r = 0.14, p = 0.28 | r = -0.03, p = 0.42 | r = -0.03, p = 0.42 |
| **CON** | r = -0.12, p = 0.30 | r = -0.08, p = 0.35 | r = 0.05, p = 0.40 |
| **DMN** | r = -0.09, p = 0.32 | r = -0.16, p = 0.23 | r = -0.01, p = 0.48 |
| **TPN** | r = -0.10, p = 0.32 | r = -0.12, p = 0.30 | r = 0.09, p = 0.32 |

**Table S2. ROI names and their functional network assignments based on the Yeo 17-network parcellation.**

| **NO** | **ROI_name** | **NO** | **ROI_name** |
| --- | --- | --- | --- |
| 1 | LH_VisCent_Striate | 38 | LH_ContB_IPL |
| 2 | LH_VisCent_ExStr | 39 | LH_ContB_PFCd |
| 3 | LH_VisPeri_Striate | 40 | LH_ContB_PFCl |
| 4 | LH_VisPeri_ExStrInf | 41 | LH_ContB_PFClv |
| 5 | LH_VisPeri_ExStrSup | 42 | LH_ContB_PFCmp |
| 6 | LH_SomMotA | 43 | LH_ContC_pCun |
| 7 | LH_SomMotB_Cent | 44 | LH_ContC_Cingp |
| 8 | LH_SomMotB_S2 | 45 | LH_DefaultA_IPL |
| 9 | LH_SomMotB_Ins | 46 | LH_DefaultA_PFCd |
| 10 | LH_SomMotB_Aud | 47 | LH_DefaultA_PCC |
| 11 | LH_DorsAttnA_TempOcc | 48 | LH_DefaultA_PFCm |
| 12 | LH_DorsAttnA_ParOcc | 49 | LH_DefaultB_Temp |
| 13 | LH_DorsAttnA_SPL | 50 | LH_DefaultB_IPL |
| 14 | LH_DorsAttnB_TempOcc | 51 | LH_DefaultB_PFCd |
| 15 | LH_DorsAttnB_PostC | 52 | LH_DefaultB_PFCl |
| 16 | LH_DorsAttnB_FEF | 53 | LH_DefaultB_PFCv |
| 17 | LH_DorsAttnB_PrCv | 54 | LH_DefaultC_IPL |
| 18 | LH_SalVentAttnA_ParOper | 55 | LH_DefaultC_Rsp |
| 19 | LH_SalVentAttnA_PrCv | 56 | LH_DefaultC_PHC |
| 20 | LH_SalVentAttnA_Ins | 57 | LH_TempPar |
| 21 | LH_SalVentAttnA_ParMed | 58 | RH_VisCent_Striate |
| 22 | LH_SalVentAttnA_FrMed | 59 | RH_VisCent_ExStr |
| 23 | LH_SalVentAttnB_IPL | 60 | RH_VisPeri_Striate |
| 24 | LH_SalVentAttnB_PFCd | 61 | RH_VisPeri_ExStrInf |
| 25 | LH_SalVentAttnB_PFCl | 62 | RH_VisPeri_ExStrSup |
| 26 | LH_SalVentAttnB_PFCv | 63 | RH_SomMotA |
| 27 | LH_SalVentAttnB_OFC | 64 | RH_SomMotB_Cent |
| 28 | LH_SalVentAttnB_PFCmp | 65 | RH_SomMotB_S2 |
| 29 | LH_Limbic_TempPole | 66 | RH_SomMotB_Ins |
| 30 | LH_Limbic_OFC | 67 | RH_SomMotB_Aud |
| 31 | LH_ContA_Temp | 68 | RH_DorsAttnA_TempOcc |
| 32 | LH_ContA_IPS | 69 | RH_DorsAttnA_ParOcc |
| 33 | LH_ContA_PFCd | 70 | RH_DorsAttnA_SPL |
| 34 | LH_ContA_PFCl | 71 | RH_DorsAttnB_TempOcc |
| 35 | LH_ContA_PFClv | 72 | RH_DorsAttnB_PostC |
| 36 | LH_ContA_Cinga | 73 | RH_DorsAttnB_FEF |
| 37 | LH_ContB_Temp | 74 | RH_DorsAttnB_PrCv |
| 75 | RH_SalVentAttnA_ParOper | 95 | RH_ContB_Temp |
| 76 | RH_SalVentAttnA_PrC | 96 | RH_ContB_IPL |
| 77 | RH_SalVentAttnA_PrCv | 97 | RH_ContB_PFCld |
| 78 | RH_SalVentAttnA_Ins | 98 | RH_ContB_PFClv |
| 79 | RH_SalVentAttnA_ParMed | 99 | RH_ContB_PFCmp |
| 80 | RH_SalVentAttnA_FrMed | 100 | RH_ContC_pCun |
| 81 | RH_SalVentAttnB_IPL | 101 | RH_ContC_Cingp |
| 82 | RH_SalVentAttnB_PFCd | 102 | RH_DefaultA_Temp |
| 83 | RH_SalVentAttnB_PFCl | 103 | RH_DefaultA_IPL |
| 84 | RH_SalVentAttnB_PFClv | 104 | RH_DefaultA_PFCd |
| 85 | RH_SalVentAttnB_PFCv | 105 | RH_DefaultA_PCC |
| 86 | RH_SalVentAttnB_PFCmp | 106 | RH_DefaultA_PFCm |
| 87 | RH_SalVentAttnB_Cinga | 107 | RH_DefaultB_Temp |
| 88 | RH_Limbic_TempPole | 108 | RH_DefaultB_AntTemp |
| 89 | RH_Limbic_OFC | 109 | RH_DefaultB_PFCd |
| 90 | RH_ContA_Temp | 110 | RH_DefaultB_PFCv |
| 91 | RH_ContA_IPS | 111 | RH_DefaultC_IPL |
| 92 | RH_ContA_PFCd | 112 | RH_DefaultC_Rsp |
| 93 | RH_ContA_PFCl | 113 | RH_DefaultC_PHC |
| 94 | RH_ContA_Cinga | 114 | RH_TempPar |

# Reference

1. Yeo BT, Krienen FM, et al. The organization of the human cerebral cortex estimated by intrinsic functional connectivity. J Neurophysiol. 2011 Sep;106(3):1125-65.
2. Schaefer A, Kong R, Gordon EM, Laumann TO, Zuo XN, Holmes AJ, Eickhoff SB, Yeo BTT. Local-Global Parcellation of the Human Cerebral Cortex from Intrinsic Functional Connectivity MRI. Cereb Cortex. 2018 Sep 1;28(9):3095-3114.
